# Supplementary material for: HIVprotI: an integrated web based platform for prediction and design of HIV proteins inhibitors
Source: J Cheminform. 2018 Mar 9;10:12. doi: 10.1186/s13321-018-0266-y (PMC5845081; doi:10.1186/s13321-018-0266-y)
Supplement: Supplementary file 2 — Additional file 2. Source code of HIVProtI web server. [file 13321_2018_266_MOESM2_ESM.zip › HIVprotI_Source-code/hivprotI/weka-3-6-6/doc/weka/filters/unsupervised/instance/Resample.html]

Resample


---


|  |  |  |
| --- | --- | --- |
| **Overview** \| **Package** \| **Class** \| **Tree** \| **Deprecated** \| **Index** \| **Help** \| **Weka's home** | |  |
| **PREV CLASS**   **NEXT CLASS** | **FRAMES**    **NO FRAMES**     **All Classes** |  |
| SUMMARY: NESTED \| FIELD \| CONSTR \| METHOD | DETAIL: FIELD \| CONSTR \| METHOD |  |


---


## weka.filters.unsupervised.instance Class Resample

```
java.lang.Object
  weka.filters.Filter
      weka.filters.unsupervised.instance.Resample
```

**All Implemented Interfaces:**: java.io.Serializable, CapabilitiesHandler, OptionHandler, RevisionHandler, UnsupervisedFilter

---

``` public class Resample extends Filter implements UnsupervisedFilter, OptionHandler ```

Produces a random subsample of a dataset using either sampling with replacement or without replacement. The original dataset must fit entirely in memory. The number of instances in the generated dataset may be specified. When used in batch mode, subsequent batches are NOT resampled.


Valid options are:

```
 -S <num>
  Specify the random number seed (default 1)
```

```
 -Z <num>
  The size of the output dataset, as a percentage of
  the input dataset (default 100)
```

```
 -no-replacement
  Disables replacement of instances
  (default: with replacement)
```

```
 -V
  Inverts the selection - only available with '-no-replacement'.
```

**Version:**
:   $Revision: 5548 $

**Author:**
:   Len Trigg (len@reeltwo.com), FracPete (fracpete at waikato dot ac dot nz)

**See Also:**: Serialized Form

---

**Constructor Summary**

`Resample()`


| **Method Summary** | |
| --- | --- |
| `boolean` | `batchFinished()`             Signify that this batch of input to the filter is finished. |
| `void` | `createSubsampleWithoutReplacement(java.util.Random random, int origSize, int sampleSize)`             creates the subsample without replacement |
| `void` | `createSubsampleWithReplacement(java.util.Random random, int origSize, int sampleSize)`             creates the subsample with replacement |
| `Capabilities` | `getCapabilities()`             Returns the Capabilities of this filter. |
| `boolean` | `getInvertSelection()`             Gets whether selection is inverted (only if instances are drawn WIHTOUT replacement). |
| `boolean` | `getNoReplacement()`             Gets whether instances are drawn with or without replacement. |
| `java.lang.String[]` | `getOptions()`             Gets the current settings of the filter. |
| `int` | `getRandomSeed()`             Gets the random number seed. |
| `java.lang.String` | `getRevision()`             Returns the revision string. |
| `double` | `getSampleSizePercent()`             Gets the subsample size as a percentage of the original set. |
| `java.lang.String` | `globalInfo()`             Returns a string describing this classifier |
| `boolean` | `input(Instance instance)`             Input an instance for filtering. |
| `java.lang.String` | `invertSelectionTipText()`             Returns the tip text for this property |
| `java.util.Enumeration` | `listOptions()`             Returns an enumeration describing the available options. |
| `static void` | `main(java.lang.String[] argv)`             Main method for testing this class. |
| `java.lang.String` | `noReplacementTipText()`             Returns the tip text for this property |
| `java.lang.String` | `randomSeedTipText()`             Returns the tip text for this property |
| `java.lang.String` | `sampleSizePercentTipText()`             Returns the tip text for this property |
| `boolean` | `setInputFormat(Instances instanceInfo)`             Sets the format of the input instances. |
| `void` | `setInvertSelection(boolean value)`             Sets whether the selection is inverted (only if instances are drawn WIHTOUT replacement). |
| `void` | `setNoReplacement(boolean value)`             Sets whether instances are drawn with or with out replacement. |
| `void` | `setOptions(java.lang.String[] options)`             Parses a given list of options. |
| `void` | `setRandomSeed(int newSeed)`             Sets the random number seed. |
| `void` | `setSampleSizePercent(double newSampleSizePercent)`             Sets the size of the subsample, as a percentage of the original set. |

**Methods inherited from class weka.filters.Filter**

`batchFilterFile, filterFile, getCapabilities, getOutputFormat, isFirstBatchDone, isNewBatch, isOutputFormatDefined, makeCopies, makeCopy, numPendingOutput, output, outputPeek, toString, useFilter, wekaStaticWrapper`

**Methods inherited from class java.lang.Object**

`equals, getClass, hashCode, notify, notifyAll, wait, wait, wait`

**Constructor Detail**

### Resample

```
public Resample()
```


**Method Detail**

### globalInfo

```
public java.lang.String globalInfo()
```

:   Returns a string describing this classifier

    :   **Returns:**: a description of the classifier suitable for displaying in the explorer/experimenter gui

---


### listOptions

```
public java.util.Enumeration listOptions()
```

:   Returns an enumeration describing the available options.

    :   **Specified by:**: `listOptions` in interface `OptionHandler`
    :   **Returns:**: an enumeration of all the available options.

---


### setOptions

```
public void setOptions(java.lang.String[] options)
                throws java.lang.Exception
```

:   Parses a given list of options.


    Valid options are:

    ```
     -S <num>
      Specify the random number seed (default 1)
    ```

    ```
     -Z <num>
      The size of the output dataset, as a percentage of
      the input dataset (default 100)
    ```

    ```
     -no-replacement
      Disables replacement of instances
      (default: with replacement)
    ```

    ```
     -V
      Inverts the selection - only available with '-no-replacement'.
    ```

    :   **Specified by:**: `setOptions` in interface `OptionHandler`
    :   **Parameters:**: `options` - the list of options as an array of strings **Throws:**: `java.lang.Exception` - if an option is not supported

---


### getOptions

```
public java.lang.String[] getOptions()
```

:   Gets the current settings of the filter.

    :   **Specified by:**: `getOptions` in interface `OptionHandler`
    :   **Returns:**: an array of strings suitable for passing to setOptions

---


### randomSeedTipText

```
public java.lang.String randomSeedTipText()
```

:   Returns the tip text for this property

    :   **Returns:**: tip text for this property suitable for displaying in the explorer/experimenter gui

---


### getRandomSeed

```
public int getRandomSeed()
```

:   Gets the random number seed.

    :   **Returns:**: the random number seed.

---


### setRandomSeed

```
public void setRandomSeed(int newSeed)
```

:   Sets the random number seed.

    :   **Parameters:**: `newSeed` - the new random number seed.

---


### sampleSizePercentTipText

```
public java.lang.String sampleSizePercentTipText()
```

:   Returns the tip text for this property

    :   **Returns:**: tip text for this property suitable for displaying in the explorer/experimenter gui

---


### getSampleSizePercent

```
public double getSampleSizePercent()
```

:   Gets the subsample size as a percentage of the original set.

    :   **Returns:**: the subsample size

---


### setSampleSizePercent

```
public void setSampleSizePercent(double newSampleSizePercent)
```

:   Sets the size of the subsample, as a percentage of the original set.

    :   **Parameters:**: `newSampleSizePercent` - the subsample set size, between 0 and 100.

---


### noReplacementTipText

```
public java.lang.String noReplacementTipText()
```

:   Returns the tip text for this property

    :   **Returns:**: tip text for this property suitable for displaying in the explorer/experimenter gui

---


### getNoReplacement

```
public boolean getNoReplacement()
```

:   Gets whether instances are drawn with or without replacement.

    :   **Returns:**: true if the replacement is disabled

---


### setNoReplacement

```
public void setNoReplacement(boolean value)
```

:   Sets whether instances are drawn with or with out replacement.

    :   **Parameters:**: `value` - if true then the replacement of instances is disabled

---


### invertSelectionTipText

```
public java.lang.String invertSelectionTipText()
```

:   Returns the tip text for this property

    :   **Returns:**: tip text for this property suitable for displaying in the explorer/experimenter gui

---


### getInvertSelection

```
public boolean getInvertSelection()
```

:   Gets whether selection is inverted (only if instances are drawn WIHTOUT
    replacement).

    :   **Returns:**: true if the replacement is disabled **See Also:**: `m_NoReplacement`

---


### setInvertSelection

```
public void setInvertSelection(boolean value)
```

:   Sets whether the selection is inverted (only if instances are drawn WIHTOUT
    replacement).

    :   **Parameters:**: `value` - if true then selection is inverted

---


### getCapabilities

```
public Capabilities getCapabilities()
```

:   Returns the Capabilities of this filter.

    :   **Specified by:**: `getCapabilities` in interface `CapabilitiesHandler` **Overrides:**: `getCapabilities` in class `Filter`
    :   **Returns:**: the capabilities of this object **See Also:**: `Capabilities`

---


### setInputFormat

```
public boolean setInputFormat(Instances instanceInfo)
                       throws java.lang.Exception
```

:   Sets the format of the input instances.

    :   **Overrides:**: `setInputFormat` in class `Filter`
    :   **Parameters:**: `instanceInfo` - an Instances object containing the input instance structure (any instances contained in the object are ignored - only the structure is required). **Returns:**: true if the outputFormat may be collected immediately **Throws:**: `java.lang.Exception` - if the input format can't be set successfully

---


### input

```
public boolean input(Instance instance)
```

:   Input an instance for filtering. Filter requires all
    training instances be read before producing output.

    :   **Overrides:**: `input` in class `Filter`
    :   **Parameters:**: `instance` - the input instance **Returns:**: true if the filtered instance may now be collected with output(). **Throws:**: `java.lang.IllegalStateException` - if no input structure has been defined

---


### batchFinished

```
public boolean batchFinished()
```

:   Signify that this batch of input to the filter is finished.
    If the filter requires all instances prior to filtering,
    output() may now be called to retrieve the filtered instances.

    :   **Overrides:**: `batchFinished` in class `Filter`
    :   **Returns:**: true if there are instances pending output **Throws:**: `java.lang.IllegalStateException` - if no input structure has been defined

---


### createSubsampleWithReplacement

```
public void createSubsampleWithReplacement(java.util.Random random,
                                           int origSize,
                                           int sampleSize)
```

:   creates the subsample with replacement

    :   **Parameters:**: `random` - the random number generator to use: `origSize` - the original size of the dataset: `sampleSize` - the size to generate

---


### createSubsampleWithoutReplacement

```
public void createSubsampleWithoutReplacement(java.util.Random random,
                                              int origSize,
                                              int sampleSize)
```

:   creates the subsample without replacement

    :   **Parameters:**: `random` - the random number generator to use: `origSize` - the original size of the dataset: `sampleSize` - the size to generate

---


### getRevision

```
public java.lang.String getRevision()
```

:   Returns the revision string.

    :   **Specified by:**: `getRevision` in interface `RevisionHandler` **Overrides:**: `getRevision` in class `Filter`
    :   **Returns:**: the revision

---


### main

```
public static void main(java.lang.String[] argv)
```

:   Main method for testing this class.

    :   **Parameters:**: `argv` - should contain arguments to the filter: use -h for help


---


|  |  |  |
| --- | --- | --- |
| **Overview** \| **Package** \| **Class** \| **Tree** \| **Deprecated** \| **Index** \| **Help** \| **Weka's home** | |  |
| **PREV CLASS**   **NEXT CLASS** | **FRAMES**    **NO FRAMES**     **All Classes** |  |
| SUMMARY: NESTED \| FIELD \| CONSTR \| METHOD | DETAIL: FIELD \| CONSTR \| METHOD |  |


---
